# Fabrication of “electroactive cells” using bio-inspired polydopamine-derived carbon nanoparticles for manipulation of cells with electrical stimulation

**DOI:** 10.3389/fbioe.2022.949308

**Published:** 2022-07-25

**Authors:** Fang-Yi Li, Yi-Chang Chung

**Affiliations:** ^1^ Department of Chemical and Materials Engineering, National University of Kaohsiung, Kaohsiung, Taiwan; ^2^ Research Center of Biomimetics and Medicare Technology, National University of Kaohsiung, Kaohsiung, Taiwan

**Keywords:** carbon nanoparticles (CNPs), polydopamine (PDA), cell manipulation, electroactive cells, fluorescence, electronic stimulation

## Abstract

In this study, we report some bio-inspired carbon nanoparticles (CNPs) that exhibit high fluorescence quantum yields, good conductivity, excellent dispersion in aqueous solution, high cell-uptake efficiency, and no cytotoxicity as well. We were inspired by mussels’ adhesive components to synthesize polydopamine nanoparticles and then use a carbonization process to prepare fluorescent CNPs. Using some surfactants, we could control the sizes of CNPs and increase their dispersion in water. Fluorescence spectroscopy confirmed the excitation of CNPs at 360 nm and emission of blue light with a 400–450 nm wavelength. High quantum yields of greater than 20% were also measured. Transmission electron microscopy proved that the addition of surfactants could shrink particles to several nanometers in size. The fluorescent and conductive CNPs were applied to stain L929 fibroblast cells *in vitro*, finding no harmful effects on cells. Due to the polydopamine-derived CNPs’ good electrical, fluorescent, and biocompatible response, we designed a platform to manipulate the cells after endocytosis of conductive CNPs to observe the effects of electrical stimulation on cell attachment, cell growth, and cell death. The nanoparticles endocytosed by cells seemed more easily attracted to the electric field, leading to enhanced cell attachment and growth. Therefore, CNP uptake can increase the attachment of cells onto a conductive plate electrode in a short time (within 10 min at 4°C). When the source of the electric field was changed to rod electrodes in the medium, cells that had been pre-adsorbed onto a non-conductive plate were desorbed from the plate and destroyed. Therefore, addition of CNPs during cell incubation can allow control of cell growth and death via manipulation of electric fields.

## Introduction

Carbon nanoparticles (CNPs) or carbon nanodots are extremely useful in many applications, including metal ion sensing (Gao et al., 2016; Torres Landa et al., 2022), diagnostic probes (Bhunia et al., 2013; Yan et al., 2013; Yu et al., 2022), nontoxic labeling (Das et al., 2019), drug carriers (Wang et al., 2013), hydrogen generation (Jana et al., 2021; Zhao et al., 2021), specific catalysts (Makama, Umar and Saidu, 2018), and so on. Due to their graphite-like multilayers (Oza et al., 2016), nanoscale sizes, and fluorescence, CNPs may also be useful in biomedical applications to replace other fluorescent dyes. Commonly-used fluorescent particle categories include semiconductors, quantum dots, metal nanoparticles, organic nanoparticles, and so on. Most research is focused on inorganic fluorescent nanoparticles; however, some so-called quantum dots contain heavy metal ions, other toxic elements, and non-degradable crystals, so scientists have been developing some alternatives (Liu et al., 2019; M.; May et al., 2022). Additionally, inert metal nanoparticles are always expensive and easily accumulate in the body. As a result, novel CNPs have been developed by dehydrating various organic compounds to achieve fluorescence using simple synthesis routes, nontoxic materials, and inexpensive processes (Lim, Shen and Gao, 2015; Azam, Najabat Ali and Javaid Khan, 2021).

Dopamine (DA) is a hormone and neurotransmitter that plays a number of important roles in the human brain and body. It also plays an important role in marine mussel organisms as a strong adhesion group in secreted proteins. Its chemical structure contains both a catechol group and a primary amine group under basic conditions, so it is able to undergo self-polymerization to form polydopamine (PDA) nanoparticles (Farnad, Farhadi and Voelcker, 2012; Zhang et al., 2012; El Yakhlifi and Ball, 2020). Due to the universal adhesion of catechol groups and the excellent biocompatibility of its polymers, PDA has been studied extensively as a widely-used coating in various applications. Some studies have also proven that PDA formed in alkaline environments with different pH values has useful properties for many purposes, including surface modifications, nanoparticle synthesis, as a drug carrier, and so on (Liu, Ai and Lu, 2014; Lee, Park and Lee, 2020). PDA molecules with aromatic rings in a polymer chain induce π-π stacked arrays. After dehydration, PDA-based CNPs can generate some conjugate effects, resulting in a special property of electrical conductivity. Several studies have proposed the oxidation of dopamine into PDA, the structures of which are mainly composed of dihydroxyindole, indoledione, and dopamine units. Yu et al. proposed a self-assembled supramolecular structure of PDA via carbonization of PDA nanoparticles at 800°C in nitrogen (Yu et al., 2014). We further conceptualized that the carbonized PDA with six-membered and five-membered rings in a polymer structure provides carbon and nitrogen sources and a tendency to curl during dehydration of CNPs in a phosphate dehydration agent, generating highly efficient fluorescence, good electrical conductivity, and excellent dispersion in aqueous solutions as well. N-doping and N source introduction are the most-used strategies to enhance the emission of CNPs by inducing an upward shift in the Fermi level and electrons in the conduction band (Ayala et al., 2010). The amine groups (especially primary amine) in the N source (PDA in our study) can supply doping precursors and surface-passivating agents for the CNPs, which enhanced the fluorescent performance (Park et al., 2016; Qi et al., 2021).

As for the endowed conductivity of CNPs, carbon nanomaterials are known to have excellent electrical conductivity and therefore have been utilized in some solar cells, coatings of microelectrodes, transparent conductive films, electrochemical sensors, and so on. However, we found only a few publications discussing the electrical conductivity of carbon nanoparticles in biomedical applications, compared to 2D graphene nanoparticles and 1D carbon nanotubes (Campuzano, Yáñez-Sedeño and Pingarrón, 2019; Cruz-Delgado et al., 2021). Most research is focused on carbon nanotubes and graphene nanoparticles with excellent electrical conductivity, which always suffer from the dispersion issue, especially in a matrix or an aqueous solution, yet the CNPs synthesized or dehydrated in the solution state can be easily dispersed in an aqueous solution. PDA nanoparticles are electrically conductive, and we carbonized them to fabricate conjugated aromatic structures, which contributed to the conductivity.

For the consideration of increasing biocompatibility and dispersity of CNPs for cell uptake, phospholipids could be employed as a natural surfactant to encapsulate the CNPs based on the design of liposomes for use in drug delivery. Since the CNPs have good conductivity and dispersion in aqueous solutions, they display superior C/N ratios and indole-indole stacking after carbonization of PDA. Accordingly, we developed the first-ever “CNP-doped electroactive cells” and contributed some innovative work to manipulate cells under electrical fields.

## Materials and methods

*Preparation of polydopamine nanoparticles*: A tris-buffer solution (pH 10.5) was formulated to assist in self-polymerization of dopamine. A PC surfactant was added to the solution during PDA formation. Magnetic stirring was carried out vigorously prior to addition of dopamine hydrochloride in a tris-buffer solution, and stirring was continued for 3 h. Potassium dihydrogen phosphate was added to the solution and adjusted to 0.1 M. The solution was placed in a commercial 800 W microwave oven and irradiated for 15 min to dry. The solution in the vessel was rinsed with 100 ml ethanol and dried three times in order to remove unreacted dopamine. Finally, the solution was filtered using a 0.22 μm filter and then dried to form a powder.

### Cell manipulation test: materials and preparation

CNPs were quantitatively diluted to 0.4 wt% in phosphate buffer solution (PBS), filtered through a 0.22 μm needle-type filter, and irradiated with sterilizing UV light for 30 min to eliminate all infection and contamination. Mouse L929 fibroblast cells used for co-culture with CNPs were provided by the Food Industry Research and Development Institute of Taiwan and were cultured to a certain amount using the medium of 90% Alpha MEM (Minimum Essential Medium) and 10% horse serum every 3 days for measurements in the next step.

### Cell viability tests

1 ml cell suspension of L929 fibroblast cells with cell density 1×10^5^ cell/ml was poured into a 24-well culture plate for 24 ± 2 h with 5% CO_2_ blowing at 37 ± 1°C. The culture medium was then removed and rinsed with PBS solution prior to the complete attachment of cells on the culture plate surfaces. For each well, 2 ml fresh medium and 500 μL of 4 wt% CNPs in PBS were added to co-culture for 24 h. The co-culture medium was then withdrawn and the fresh MTT reagent (1 mg/ml in PBS) was added to react with living cells with 5% CO_2_ blowing at 37°C for 2 h. DMSO solution was used to dissolve purple crystalline complex which transformed from the MTT reagent via reaction with nuclei of living cells. Measurement of adsorption at 570 nm was performed for each sample using a microplate reader (BioTek, Synergy HT).

### Fluorescence images of cells via CNP staining at 37 °C

A 2 cm × 2 cm cleaned glass slide was placed into a well of a 6-well culture plate and 1 ml of L929 fibroblast cells with 1×10^5^ cell/ml was dropped into the well. After 12 h incubation for cell attachment on the glass surface, 4 ml of either FITC or CNPs (CNP, d-CNP1, or d-CNP10, 0.4 wt% in the medium) was added to co-culture with attached cells with 5% CO_2_ blowing at 37°C for a certain time. Afterward, the glass slide was rinsed with sterilized PBS and covered with transparent acrylic glue to encapsulate the sample and analyzed via a sub-confocal fluorescence microscope (Zeiss, ApoTome).

### Fluorescence images of cells via CNP staining at 4°C

Similarly to the above process, a 2 cm × 2 cm cleaned glass slide was placed into a well of a 6-well culture plate and 1 ml of L929 fibroblast cells with 1×10^5^ cell/ml was dropped into the well. After 12 h incubation for cell attachment on the glass surface, the culture plate was covered by an HDPE seal and placed in a 4°C refrigerator for 30 min in order to render cells dormant. 1 ml of FITC or CNPs (0.4 wt% in the medium) was added to co-culture with attached cells with 5% CO_2_ blowing for various incubation periods (10 min, 30 min, 2 h, and 4 h). The glass slide was rinsed with PBS, encapsulated with acrylic glue, and analyzed via the sub-confocal fluorescence microscope.

### Electrical stimulation tests

Three types of stimulation under a mild electrical field were carried out: (1) endocytosed cells for attachment on an electrified conductive plate; (2) viability and growth measurement of CNPs-endocytosis cells on an electrified substrate; (3) detachment and apoptosis of attached cells after endocytosis via a pair of electrified electrodes. All the detailed setups and measurements can be read in the supporting information.

## Results and discussion

In the preparation of highly-dispersive CNPs, we added phosphatidylcholine to the solution to coat the PDA and restrict the size of PDA nanoparticles. The following dehydration process was carried out via a commercial microwave oven. The microwave-assisted synthesis was found to be simple, scalable, time-saving, cost-efficient and eco-friendly compared to other preparation methods (Wang et al., 2011; Huang et al., 2015; Azam, Najabat Ali and Javaid Khan, 2021).

**FIGURE 1:**
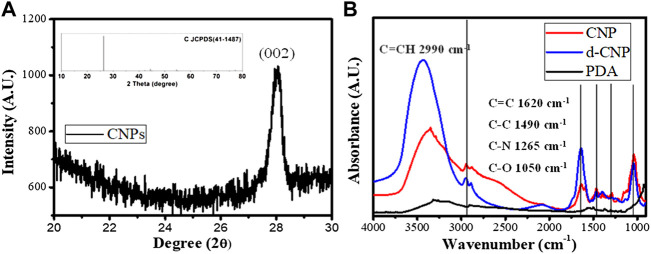
Characterization of CNPs. **(A)** XRD diffraction spectrum and the related JCPDS for carbon materials (inset). **(B)** IR spectra of CNP, d-CNP1, and PDA.

**FIGURE 2:**
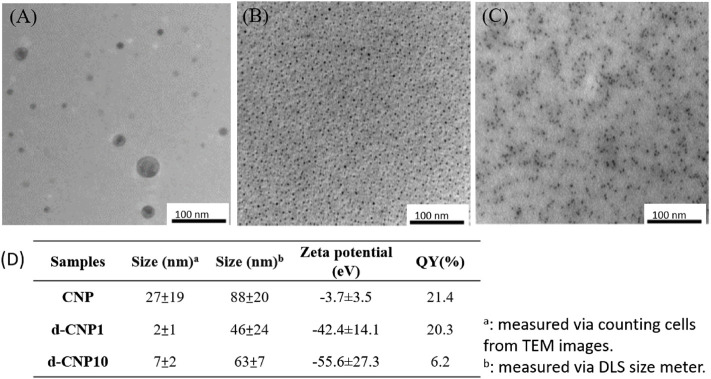
TEM images of **(A)** CNP, **(B)** d-CNP1and **(C)** d-CNP10. **(D)** Table listing the average sizes, zeta potentials, and quantum yields of CNPs.

In the characterization of CNPs, FTIR spectra showed that the carbonization of PDA led to the enhancement of C=C groups (1620 cm^−1^ for C=C stretching and 2990 cm^−1^ for C=C-H stretching) via formation of heterocyclic conjugated structures. Surfactant addition benefited the outer surface of nanoparticles containing more hydroxyl groups (around 3300 cm^−1^), resulting in good dispersion in aqueous solution. The absorption spectra of CNPs are shown in Supplementary Figure S1, revealing that the main peak of absorption caused by indole groups of PDA did not shift before or after carbonization. FTIR and UV-vis spectra showed the carbonized polydopamine nanoparticle retaining PDA structures inside. The XRD pattern of the CNPs referred to the JCPDS card no. 41-1487, illustrating that the material obtained is a graphite carbon material with a lattice of (002). Nitrogen has higher electronegativity than carbon, and the atomic radius is slightly smaller than carbon. Therefore, the N-doped or N-containing carbon materials shrink the crystal plane and reduce interplanar spacing, revealing 2θ shifts to the right (∼28^o^). Therefore, the CNPs displayed excellent conductivity, even better than commercial carbon tape (shown in Supplementary Figure S1). TEM analysis shows the excellent dispersion property of d-CNP when coated with a surfactant, compared to the TEM photo in the literature, which shows worm-like nanoparticles tens of nanometers in diameter and several hundred nanometers in length (Zhang et al., 2012). The carbonization process greatly reduced the size of the CNPs while preserving the PC surfactant coating. The deviation in nanoparticle size could also be reduced. Another size analysis using a Zetasizer 2000 determined the hydrodynamic diameters of the nanoparticles, showing that addition of the surfactant could reduce the particle size by half. However, addition of 10 times the surfactant concentration (d-CNP10) resulted in reduced particle size but also caused agglomeration due to encapsulation in excess surfactant. All the d-CNP revealed high negative zeta potentials, producing an electrostatic effect and leading to excellent dispersion in water. The characterization of CNPs is shown in Figure 1. Theoretically, after formation of polydopamine by self-polymerization via oxidation in an alkaline solution, the PDA was encapsulated by the surfactant micelles immediately and thus limited in size (Zhang et al., 2014). Due to CNPs being prepared by heating in a microwave oven, the original PDA maintained its consecutive conjugated double bonds and was completely oxidized during this process. The fluorescence quantum yields of our CNPs were relatively high compared to CNPs formed from polymers containing long carbon chains, as shown in the table in Figure 2.

**FIGURE 3:**
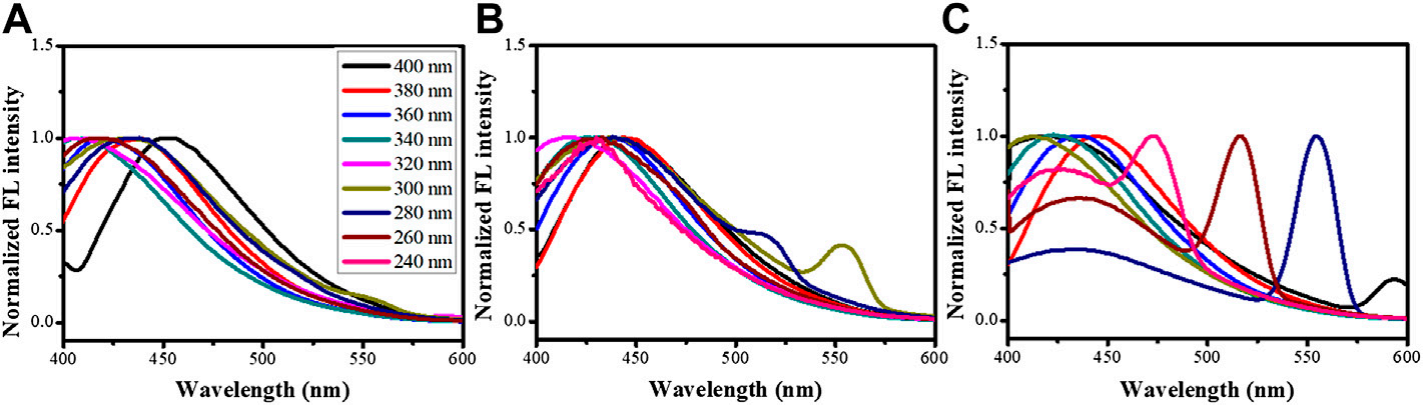
Fluorescence emission spectra of **(A)** CNP, **(B)** d-CNP1, and **(C)** d-CNP10, under excitation lights with various wavelengths.

The CNPs were measured for their fluorescent property using 220-400-nm-wavelength UV light for excitation, as can be seen in Figure 3. Fluorescent spectra showed that CNP and d-CNP1 displayed high fluorescent intensity at around 400–450 nm regions, which is blue light. Comparing the various excitation wavelengths, both CNP and d-CNP1 showed the highest efficiency at 360 nm, while the d-CNP10 performed at 340 nm and looked blue-green at the short excitation wavelength lights due to its excitation frequency multiplication. This indicated the relatively low fluorescent intensity for d-CNP10, which contained more PC surfactants. The quantum yields (QY) of samples were measured for their absorption and emission relationships with a standard quinine hemisulfate, which displays the highest fluorescent intensity at 365 nm, similar to CNPs (Wang et al., 2011). All the experimental details and the spectra are shown in the supporting information. The interesting results indicated that high quantum yields of nanoparticles could be found: CNP, d-CNP1, and d-CNP10 with 21.4, 20.3 and 6.2% QY, respectively. In comparison with organic fluorescent dyes and inorganic quantum dots, the CNPs display high QY and low bleaching effect with a different synthesis process and flexible adjustment via different carbon resources (Wang and Hu, 2014). Addition of surfactants caused good dispersion but might hinder the electron conjugation of heterocyclic structures in PC micelles, indicating a slight decrease in QY for low surfactant concentrations and a severe decrease in QY for high surfactant concentrations.

**FIGURE 4:**
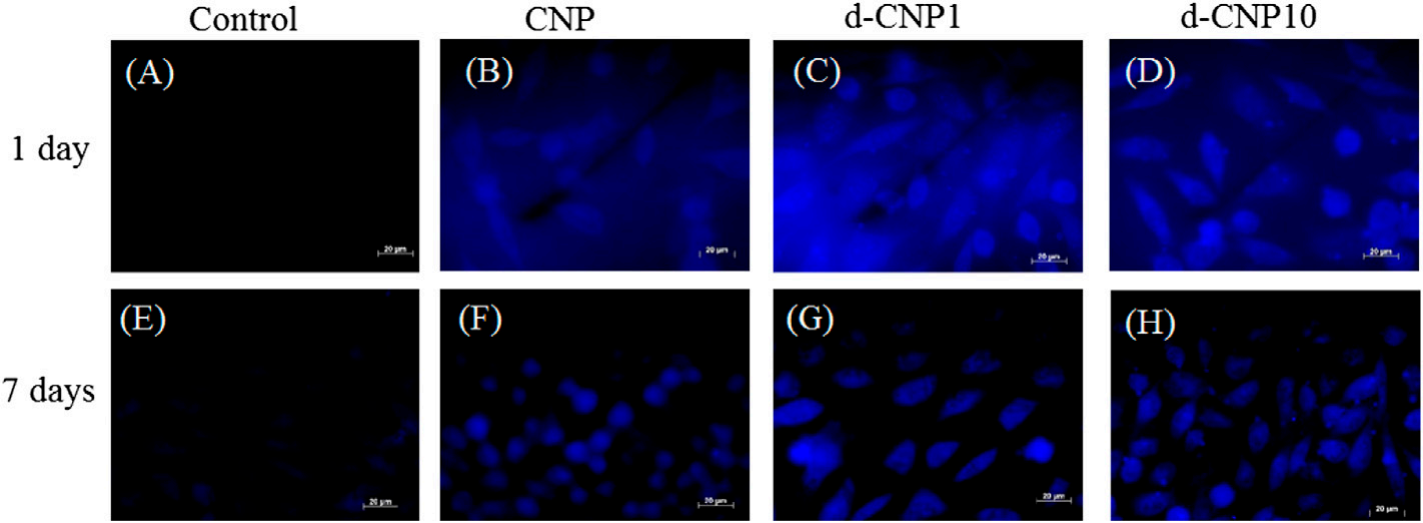
Fluorescent images of L929 cells incubated with CNPs for 1 day and 1 week. **(A)**, **(E)**: blank; **(B)**, **(F)**: CNP; **(C)**, **(G)**: d-CNP1; **(D)**, **(H)**: d-CNP10. Scale bar: 20 μm.

The high QY effect benefited the staining and observation of cell cultures. We co-incubated L929 fibroblast cells with CNPs for various periods of time at 4 and 37°C in order to observe the differences between FITC and various CNPs. Figure 4 shows the confocal microphotographs of cells at the normal incubation temperature of 37°C for different periods. FITC (1 mg/ml) with a high QY of 92% can be used as a reference for staining on cell membranes between 12–24 h of incubation (Lynge et al., 2011). We observed the weak autofluorescence of cells (blank) under irradiation of 365 nm light in photos (A) and (E), revealing significantly different images for CNP-dyed cells. Especially with PC surfactants, the d-CNP series exhibited a better staining effect on cell skeletons, plasmins, membranes, and even nuclei. Seven-day incubation was employed to explore the uptake of CNPs in the cell subculture, and all the CNPs were found to show a long-term fluorescent property and to be transferred to the next generation of cells. The sizes of cells on Day 7 are the same as on Day 1, but they look a little smaller due to the reflected fluorescence of cells in the dark fields. The phenomenon can be explained as the dilution of CNPs by the medium and newly proliferated cells during 7-days incubation, leading to fewer CNPs distributed in the cell interiors and weaker fluorescence. From photo series C and D in Figure 4, we can also see the excellent adhesion on culture flasks for cell uptake of d-CNPs, probably due to even dispersion and small nanoparticles inducing cell stretching and movement. Another short-term (6 h) incubation test, shown in Supplementary Figure S4, was performed to compare the effect of staining. FITC was found to dye only a few cells due to poor labeling efficiency, while the nano-sized CNPs could enter cell interiors in a short time. Interestingly, d-CNP1 showed complete cell images after 6 h of incubation, probably owing to phospholipid shells of CNPs enhancing the diffusion of nanoparticles (Chung, Chen and Chen, 2008; Liu et al., 2017).

**FIGURE 5:**
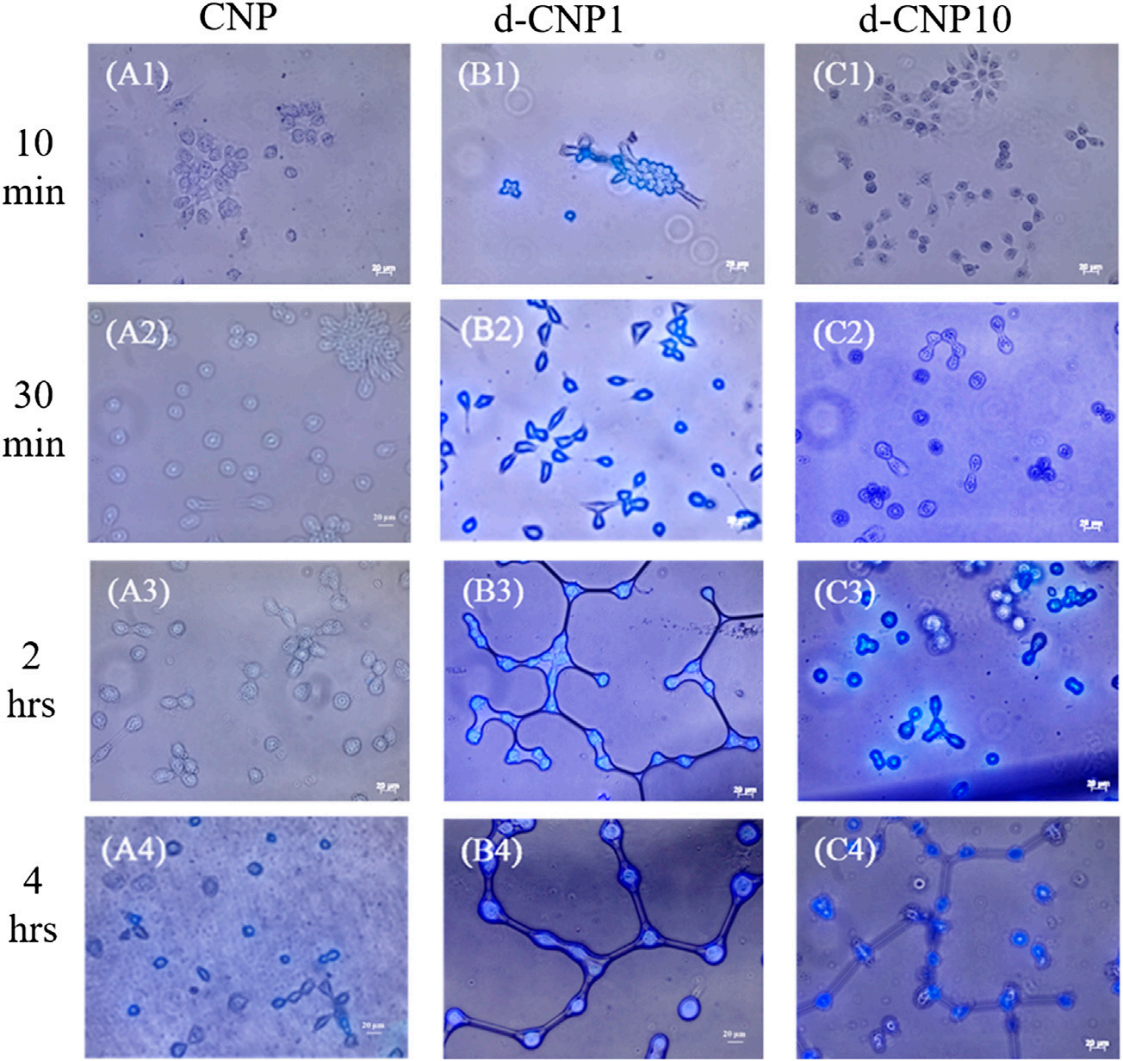
Photos of CNP-stained cells after different incubation periods at 4°C. (A1)-(A4) for CNP incubated with cells for 10 min, 30 min, 2 h and 4 h, respectively; (B1)-(B4) for d-CNP1 with those corresponding incubation periods; and (C1)-(C4) for d-CNP10 samples with corresponding periods. Scale bar: 20 μm.

In order to check the effect of PC surfactant addition, we also designed a low temperature (4°C) incubation, which basically blocked the endocytosis pathway and rendered cells dormant. The nanoparticles entering cells through direct fusion with the plasma membrane or via endocytosis can be investigated for the uptake efficiency at 37 or 4°C (Delenclos et al., 2017). In Figure 5, we can find cells stained within 10 min of incubation with d-CNPs, while the CNPs took at least 4 h to stain cells. Compared to the cell uptake with d-CNP1, the d-CNP10 containing more PC surfactants might condense and form oily gel phases at 4°C, not beneficial to the cell membrane fusion with PC-modified CNPs. As for the cytotoxicity, which correlated to the safety of using CNPs, we employed an MTT assay (ISO10993) for 24 h and even 7 days of incubation, suggesting the excellent cell compatibility of CNPs. The results show that neither CNPs nor d-CNPs revealed a significant difference in cell viability in comparison to the flask control, as shown in Supplementary Figure S3.

The PDA-derived CNPs not only demonstrated high fluorescence efficiency but also good conductivity. We designed the electrical stimulation tests for cells before and after CNP uptake. Due to electroactive CNPs being endocytosed into cells, which then become electrically-responsive cells, the cells could be stimulated and controlled via electrical fields. We examined three effects of stimulation under a mild electrical field, respectively. (1) Cell attachment tests: to locate CNP-endocytosed electroactive cells on an electrified conductive plate and to check their attachment; (2) Cell viability tests: to measure the viability and growth of CNP-endocytosed electroactive cells on an electrified substrate; (3) Induced-apoptosis tests: to explore the detachment and apoptosis of CNP-endocytosed electroactive cells via induction of a pair of electrified electrodes.

**FIGURE 6:**
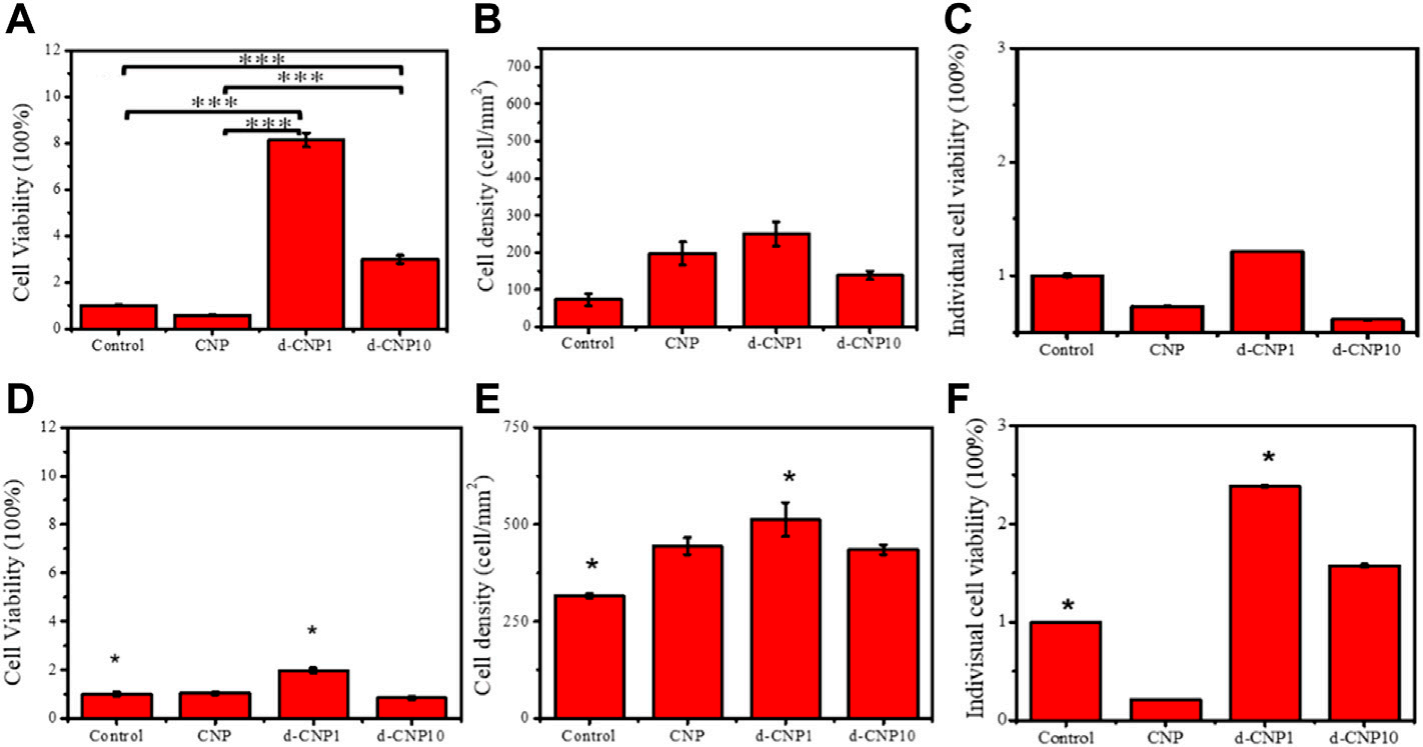
Indicators of **(A)** total cell viability, **(B)** cell attachment density, and **(C)** individual cell viability for 3 h incubation without stimulation. **(D)**–**(F)** show the same indicators for 3 h electrical stimulation. (n = 6, * represents ρ < 0.05 and *** for ρ < 0.001)

In order to investigate the cell attachment after endocytosis of CNPs on a conductive plate with mild and short-term electrical stimulation, the culture plate was modified with a sputtered gold layer on the bottom and walls, which was connected to a power supply of 3 V and 0.70 A (as shown in the schematic of Supplementary Figure S5) in the cell attachment tests. Without electrical stimulation, the CNP-endocytosed cells formed a round shape instead of a spindle shape, evidencing incomplete attachment to the gold-coated substrate (shown in Supplementary Figure S6). The total cell viability and cell attachment density were measured, and individual cell viability was then calculated from total cell viability divided by cell attachment density, which can be expressed as the viability of a single cell. Figure 6 indicates the significant differences between the d-CNP series and the control. Without stimulation, it was also obvious that CNPs led to more cell attachment than the control on the gold-coated substrate. The trend was similar to that of 3 h of electrical stimulation, where the d-CNP1-endocytosed cells showed markedly superior total viability and even individual cell viability. All the CNP-doped cells seemed to form a spindle shape, caused by firmly attaching to the substrate and stretching out their cytoskeletons, but the control remained round (Supplementary Figures S6, S7); while with 1h of stimulation and incubation, all the endocytosed cells still formed round shapes, reasonably indicating incomplete attachment and low viability.

**FIGURE 7:**
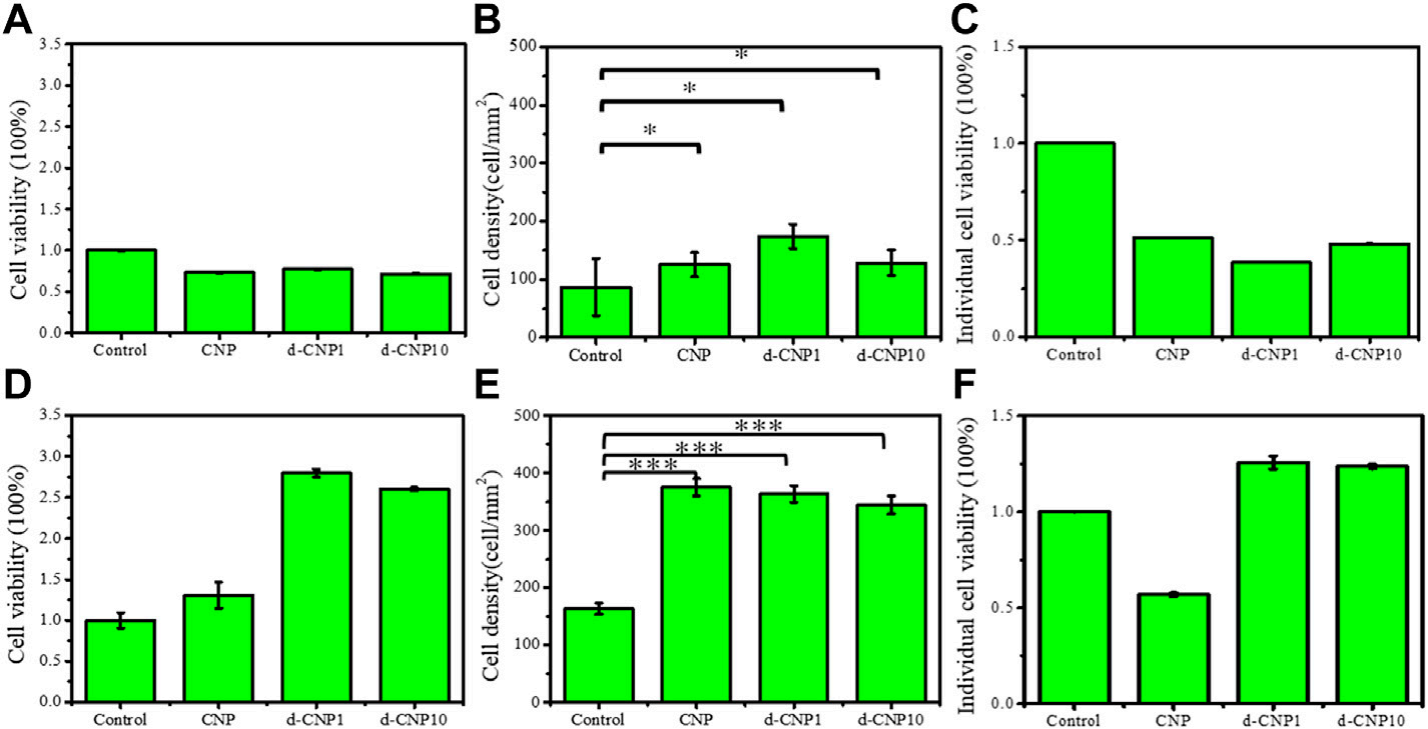
**(A)** total cell viability, **(B)** cell attachment density, and **(C)** individual cell viability for cell incubation with 3 V electrical stimulation for 1 hr. **(D)**–**(F)** show the same indicators for 4 h stimulation and incubation. (n = 6, * represents ρ < 0.05 and *** for ρ < 0.001)

In order to evaluate the cell viability and growth on an electrified substrate after endocytosis of CNPs, the cells were adhered to the substrate preferentially and then stimulated with electricity for 1 and 4 h in the cell viability tests. As can be seen in Figure 7, the initial cell attachment densities were high for CNP-doped attached cells, and the individual cell viabilities were relatively lower than the control. After 4 h of stimulation, the endocytosed cells were given enough stimulation to stretch and grow, revealing spindle shapes. Interestingly, due to the ability of the d-CNP series to penetrate through cell membranes more easily, the indicators of total cell viability, attachment density, and individual viability were better than those of CNP and control samples. After 4 h of incubation and stimulation, all the cells were treated with trypsin and encapsulated in glass slides for the observation of fluorescence dying, leading to the cells looking round. The CNP-doped cells still displayed distinct blue fluorescence (Supplementary Figure S9).

**FIGURE 8:**
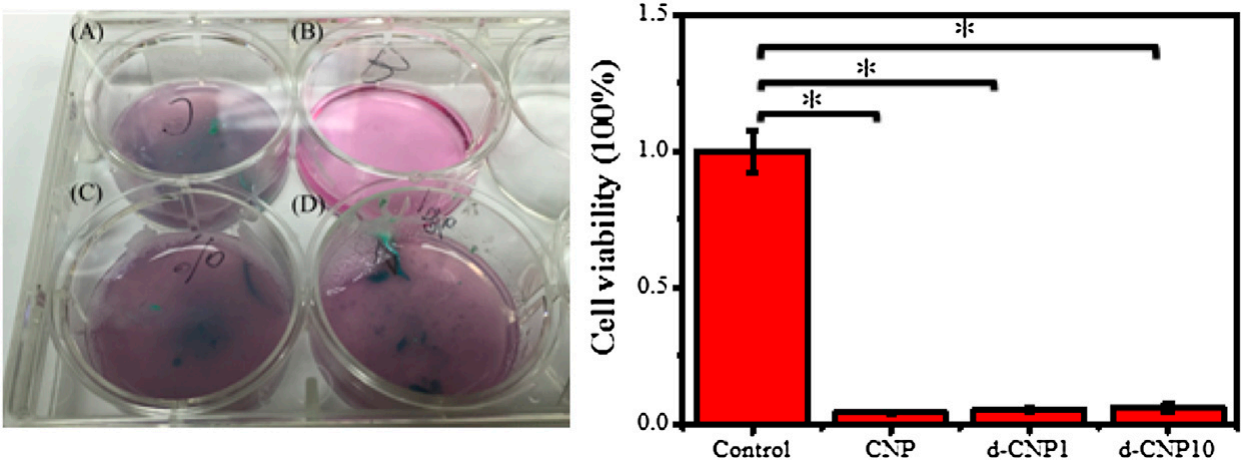
(Left) Images of pre-attached electroactive cells on a 6-well culture plate after 1 h of electrical stimulation. **(A)** CNP, **(B)** control, **(C)** d-CNP1 and **(D)** d-CNP10. (Right) The statistical cell viability for the electroactive cells after electrical stimulation. (n = 6, ρ < 0.001)

In the evaluation of detachment and apoptosis of attached cells after endocytosis via induction of a pair of electrified electrodes in the induced-apoptosis tests, we preferentially incubated cells firmly attached on general plastic culture plates and then electrified a pair of electrodes that were located in the culture medium. 3 V and 0.70 A of current were applied to the electrodes, and they were expected to show a certain attraction to those CNP-doped electroactive cells. Figure 8 shows the results of endocytosed cells after electrical stimulation, indicating few cells remained adhered to the bottom of the culture plate, while the cells of the control group still adhered to the substrate, reasonably suggesting the endocytosed cells were conductive and electrically responsive to the outer electric field. The optical photos before and after electrical stimulation are shown in Supplementary Figure S11 **.** After the stimulation, the remaining activity of endocytosed cells was only 4.4, 5.3, and 5.9% for CNP, d-CNP1, and d-CNP10, respectively, showing the lethal effect on the cells. The cells seemed to be pulled and broken from the substrate surface to the medium, causing the medium to become turbid.

For the consideration of the operating voltage effect, we also applied electrochemical scanning via the cyclic voltammetry mode under 1 V, which is similar to the scanning in electrochemistry and biosensors. In that case, we needed to take more time (24 h) to induce cell apoptosis using the induction of electrical shock (data not shown here). Furthermore, the targeting toward tumor cells could be designed according to the specific markers; for example, we have been developing another design, fabricating CNPs with folic acid to identify human breast cancer cells, which is expected to get published in the near future.

## Conclusion

We used polydopamine with five- and 6-membered consecutively conjugated cyclic structures with nitrogen doping as the precursors of carbon nanoparticles with addition of phosphatidylcholine as a surfactant to disperse the CNPs, and we processed the carbonization with a microwave oven to produce well-dispersed CNPs showing high fluorescence quantum yields and good conductivity. The CNPs were several nanometers in size, with zeta potentials surpassing -44 mV, and showed blue fluorescent light under irradiation with a 365 nm UV light. The d-CNP1 and d-CNP10 could penetrate cell interiors within 10 min at 4°C, exhibiting high staining efficiency. MTT tests demonstrated that CNPs were safe and possessed high biocompatibility without any adverse effect on cell viability. With electrical stimulation, the CNP-doped cells displayed higher cell attachment on an electrified gold-coated dish than the control. With electrical stimulation on a gold-coated Petri dish where cells were preferentially doped with CNPs and attached, the current was found to enhance the cell proliferation and activity. With the application of electrical stimulation on a pair of electrodes in a cell culture medium, the CNP-doped cells preferentially cultured on a plastic Petri dish were found to detach from the substrate surface, and at least 95% of the pre-attached cells were destroyed within 1 h. The surfactant-assisted CNPs could be dispersed well in aqueous solutions and penetrate into cells, displaying high fluorescence labeling and allowing cells to be manipulated with external electrical stimulation, suggesting a promising potential to fabricate a platform of “electroactive cells” to enhance cell attachment, to promote cell growth, and to remove harmful cells under electronic control in the future.

## Data Availability

The original contributions presented in the study are included in the article/Supplementary Material, further inquiries can be directed to the corresponding author.
