# Supplementary material for: Fabrication of “electroactive cells” using bio-inspired polydopamine-derived carbon nanoparticles for manipulation of cells with electrical stimulation
Source: Front Bioeng Biotechnol. 2022 Jul 25;10:949308. doi: 10.3389/fbioe.2022.949308 (PMC9358047; doi:10.3389/fbioe.2022.949308)
Supplement: Supplementary file 1 [file DataSheet1.docx]

**Supporting information**

Fabrication of “electroactive cells” using bio-inspired polydopamine-derived carbon nanoparticles for manipulation of cells with electrical stimulation

Fang-Yi Li ^1^, Yi-Chang Chung^1,2*^

^1^Department of Chemical and Materials Engineering, National University of Kaohsiung, Kaohsiung, 811 Taiwan Republic of China

^2^Research Center of Biomimetics and Medicare Technology, National University of Kaohsiung, Kaohsiung, 811 Taiwan Republic of China

*** Correspondence:**Yi-Chang Chung
ycchung@nuk.edu.tw

- Physical properties measurement: light absorption and conductivity measurement


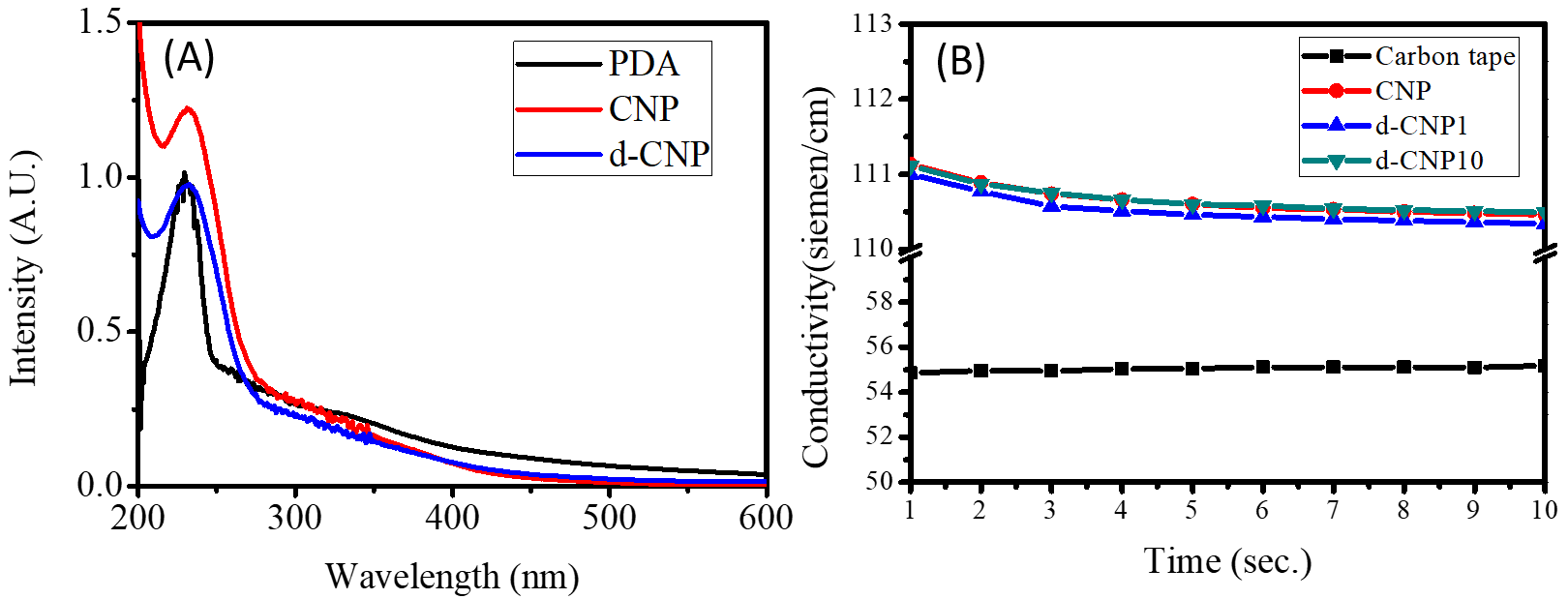


FigS1. (A) UV-vis spectra and (B) conductivity analysis of PDA, CNPs, and PC-CNPs.

- Quantum yields of CNPs

In order to estimate the quantum yield of newly-synthesized CNPs, a quinine hemisulfate solution, 2ppm in 0.1M sulfuric acid, was prepared and then diluted into 4 different concentrations for establishing a calibration curve. Using 365 nm wavelength UV light to excite the fluorescence of samples, the area of the emission curve was integrated in the fluorescent spectrum. In addition, the 4 different concentrations of quinine hemisulfate were measured for their absorption at 365 nm via UV-Vis spectraphotometer. The slope of a curve $({Grad}_{ST})$ was then estimated using the area of fluorescent curve versus absorption intensity at 365nm. The slopes of CNPs ${(Grad}_{\chi})$ were also calculated using the same method and then substituted into the Equation (1) to calculate the quantum yield of CNPs ${(\phi}_{\chi})$ as follows:

$$\phi_{\chi}=\phi_{ST}(\frac{{Grad}_{\chi}}{{Grad}_{ST}})(\frac{\varphi_{\chi}^{2}}{\varphi_{ST}^{2}})\ldots\ldots\ldots(1)$$

$\phi_{\chi}:$quantum yield of a sample; $\phi_{ST}:$ quantum yield of quinine hemisulfate (standard); ${Grad}_{\chi}:$curve slope for sample’s fluorescence area to its absorption intensity; ${Grad}_{ST}$: curve slope for a standard’s fluorescence area to its absorption intensity; $\varphi_{\chi}^{2}$: square value of a sample’s refractive index; $\varphi_{ST}^{2}$: square value of a standard’s refractive index.


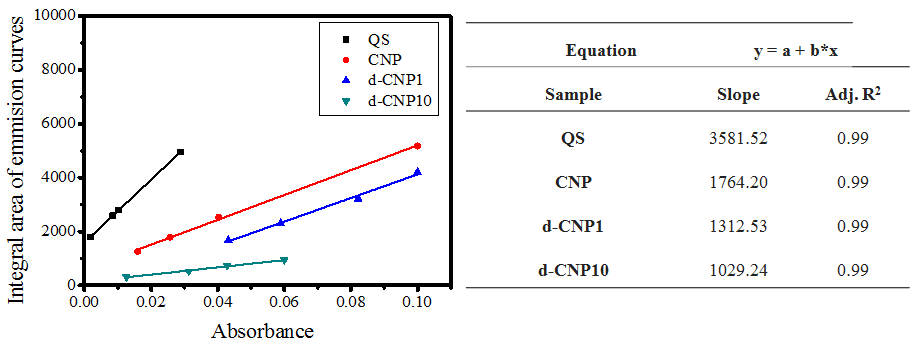


Fig. S2. Integral area of emission in fluorescence spectra versus absorbance changes for the evaluation of fluorescence efficiency.

- **In vitro cell culture measurements**

**Materials and preparation**

CNPs were quantitatively diluted to 0.4 wt% in phoshpate buffer solution (PBS) and filtered through 0.22 μm needle-type filter and irradiated with sterilizing UV light for 30 min to eliminate all infection and contamination. Mouse L929 fibroblast cells used for co-culture with CNPs were provided by the Food Industry Research and Development Institute of Taiwan, and were cultured to a certain amount using the medium of 90% Alpha MEM (Minimum Essential Medium) and 10% horse serum for every 3 days for next step measurements.

**Cell viability tests**

1 ml cell suspension with cell density 1×105 cell/ml of L929 fibroblast cells were poured into a 24- well culture plate for 24±2 hr with 5 % CO2 blowing at 37±1 oC. The culture medium was then removed and rinsed with PBS solution prior to the complete attachment of cells on the culture plate surfaces. For each well, 2 ml fresh medium and 500μl of 4 wt% CNPs in PBS were added to co-culture for 24 hr. The co-culture medium was then withdrawn and the fresh MTT reagent (1 mg/ml in PBS) was added to react with living cells with 5% CO_2_ blowing at 37 ^o^C for 2 hr. DMSO solution was used to dissolve purple crystalline complex which transformed from the MTT reagent via reaction with nuclei of living cells. Measurement of adsorption at 570 nm was performed for each sample using a microplate reader (BioTek, Synergy HT).

We cultured another set of cells similarly to the first, but left them for three days before replacing the medium with fresh horse serum containing no CNPs in order to test cell viability of the subculture after seven days. Cell viability of the subculture was tested in the same way as above.


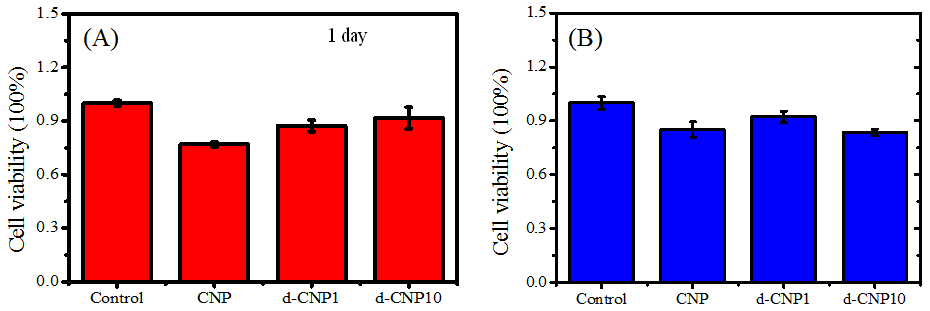


Fig. S3. MTT assay for cytotoxicity evaluation of CNP, d-CNP1, and d-CNP10 on L929 fibroblast for (A) 24 hr incubation and (B) subculture, ρ> 0.05.

**Fluorescence images of cells via CNPs staining 37 ^o^C**

A 2 cm × 2 cm cleaned glass slide was placed into a well of a 6-well culture plate and 1 ml of L929 fibroblast cells with 1×10^5^ cell/ml was dropped into the well. After 12 hr incubation for cell attachment on the glass surface, 4 ml of FITC or CNPs (CNP, d-CNP1, or d-CNP10, 0.4 wt % in the medium) was added to co-culture with attached cells with 5% CO_2_ blowing at 37 ^o^C for a certain time. Afterward, the glass slide was rinsed with sterilized PBS and covered with a transparent acrylic glue to encapsulate the sample and analyzed via a sub-confocal fluorescence microscope (Zeiss, ApoTome).


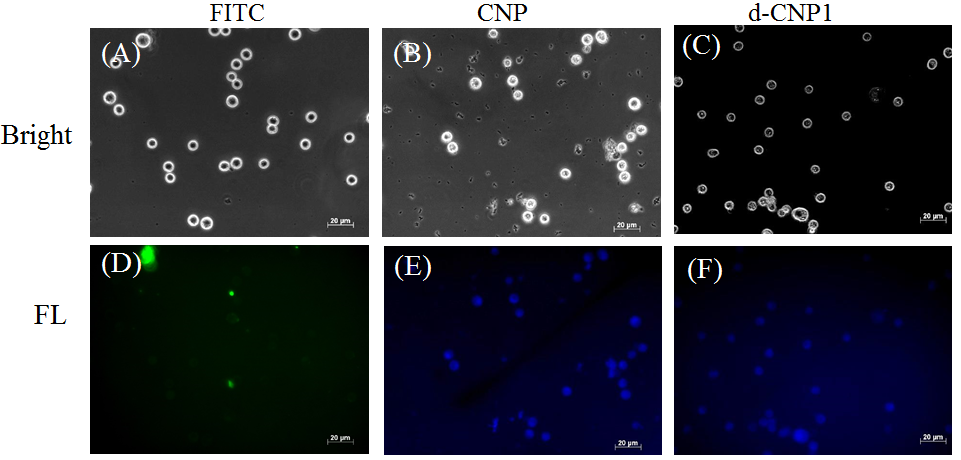


Fig. S4. Confocal microphotographs of FITC or CNPs stained for L929 cells for 6 hr. Bright field optical images for (A) FITC, (B) CNP, (C) d-CNP1 and fluorescent images for (D) FITC, (E) CNP, (F) d-CNP1.

**Fluorescence images of cells via CNP staining at 4 ^o^C**

As similar to the fluorescence staining process, a 2 cm × 2 cm cleaned glass slide was located into a well of a 6-well culture plate and 1 ml of L929 fibroblast cells with 1×10^5^ cell/ml was dropped into the well. After 12 hr incubation for cell attachment on the glass surface, the culture plate was covered by an HDPE seal and placed in the 4 ^o^C refrigerator for 30 min in order to render cells dormant. 1 ml of FITC or CNPs (0.4 wt % in the medium) was added to co-culture with attached cells with 5% CO_2_ blowing for various incubation periods (10 min, 30 min, 2 hr, and 4 hr). The glass slide was rinsed with PBS, encapsulated with acrylic glue, and analyzed via the sub-confocal fluorescence microscope.

- **Electrical stimulation tests**

Three types of stimulation under a mild electrical field: (1) endocytosed cells for attachment on an electrified conductive plate; (2) viability and growth measurement of CNPs-endocytosis cells on an electrified substrate; (3) detachment and apoptosis of attached cells after endocytosis via a pair of electrified electrodes.

(1) Endocytosed cells for attachment on an electrified conductive plate

The cell culture plate was coated with a 50 nm gold layer via sputtering process for the introduction of conductive and smooth surface for cell attachment. The 1 ml L929 fibroblast cells were then seeded into the conductive plate with 1×10^5^ cell/ml density. Cells without CNPs were used as a blank control. All the cells were incubated at 37±1 ^o^C under 5% CO_2_ blowing, with a power supply providing 3 volts and 0.70 amps, as shown in Fig. S5. The electrical power was applied for 0, 1 and 3 hr, and then the cell images were statically counted using an optical microscope focused on 10 arbitrary locations. The cells after electrical stimulation were also measured for their activity via MTT assay. The cell images before and after stimulation were shown in Fig. S6 and S7. Calculation of total cell viability and number of cell attachment and usage of Eq. 2 can reveal the individual cell viability, which might show the electrical stimulation effect on a single cell. The results are shown in the article.

$$individual cell viability=\frac{total cell viability}{cell density}\text{×100\%……………}\text{(}\text{2}\text{)}$$

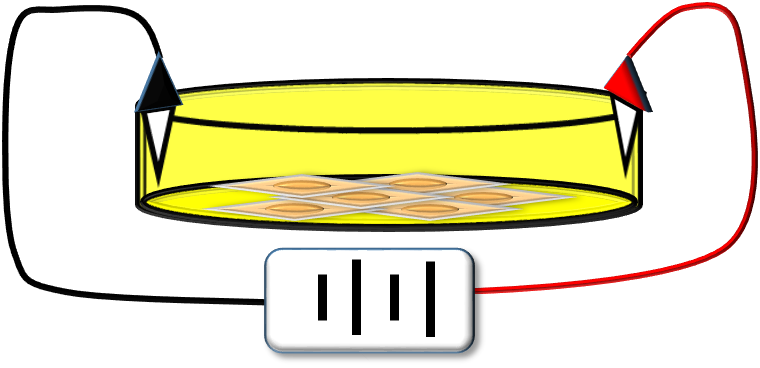


Fig. S5. Design of CNP-endocytosed cells for cell attachment tests.


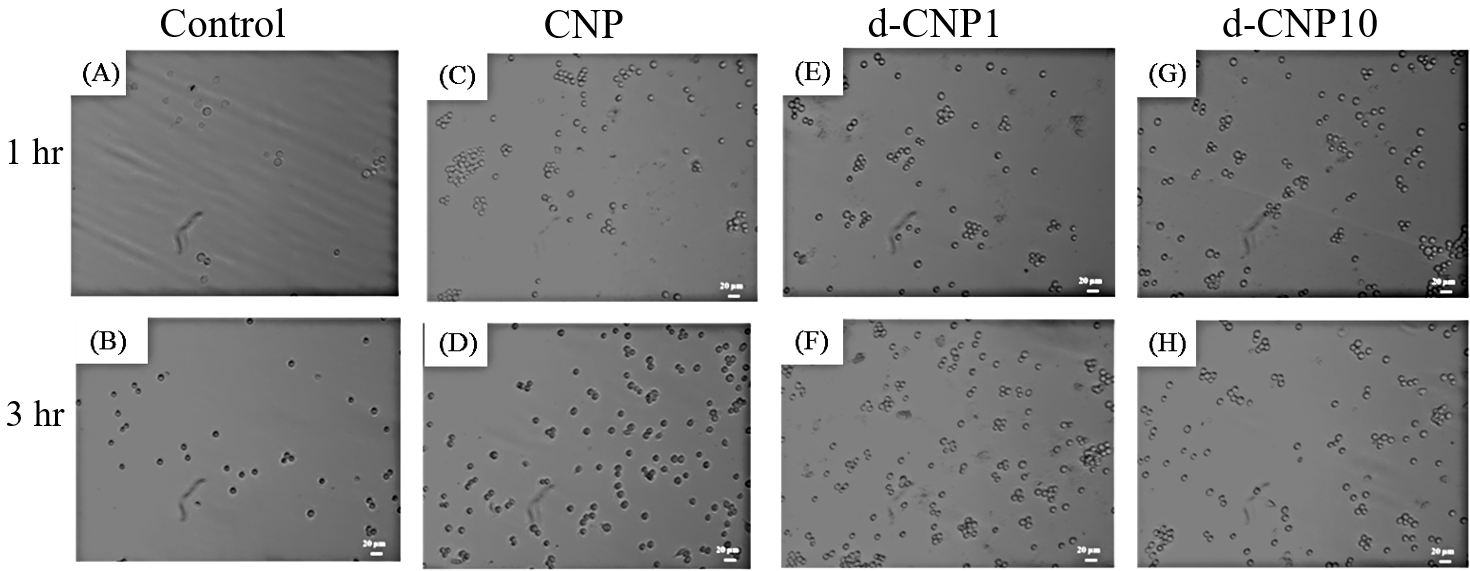


Fig. S6. Optical microphotographs (phase images) for cell attachment and growth with CNPs on a gold-coated dish with no electrical stimulation. (A) Control, (C) CNP, (E) d- CNP1, (G) d-CNP10 for 1hr of incubation and (B) control, (D) CNP, (F) d-CNP1, (H) d-CNP10 for 3hr of incubation. Scale bar: 20 μm.


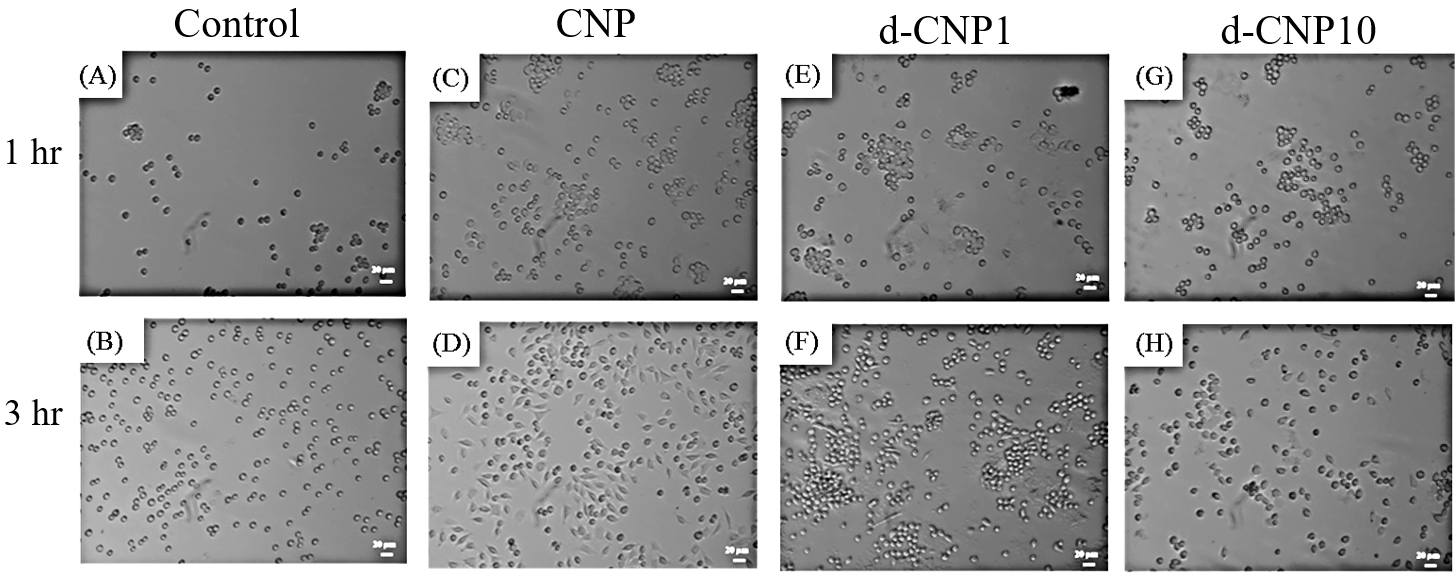


Fig. S7. Optical microphotographs (phase images) for cell attachment and growth with CNPs on a gold-coated dish with 3 V electrical stimulation. (A) Control, (C) CNP, (E) d- CNP1, (G) d-CNP10 for 1hr of incubation and (B) control, (D) CNP, (F) d-CNP1, (H) d-CNP10 for 3hr of incubation. Scale bar: 20 μm.

(2) Electroactive cell viability and growth after endocytosis on an electrified substrate.

The cell culture plate was coated with a 50 nm gold layer as mentioned above. The 1 ml L929 fibroblast cells were then seeded onto the conductive plate with 1×10^5^ cell/ml density. Cells without CNPs were used as a blank control. All the cells were incubated at 37±1 ^o^C under 5% CO_2_ blowing, with a power supply providing 3 volts, as shown in Fig. S8, for a certain time to measure the electric stimulation effect on pre-attached cells. MTT assay and optical microscopic observation were also performed to evaluate the viability of electroactive cells after short-term electrical stimulation effect.


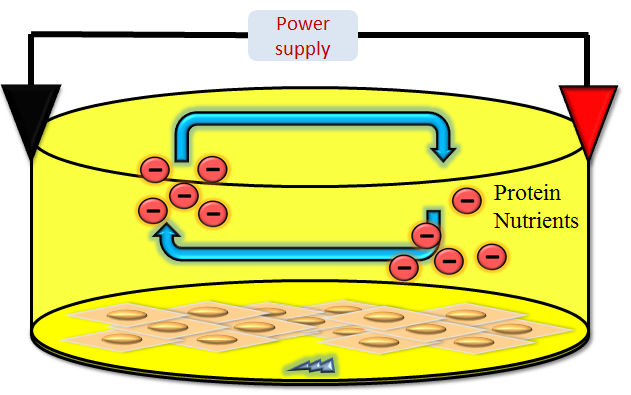


Fig.S8. Illustration of pre-attached CNP-doped electroactive cells on a conductive plate and with electrical stimulation.


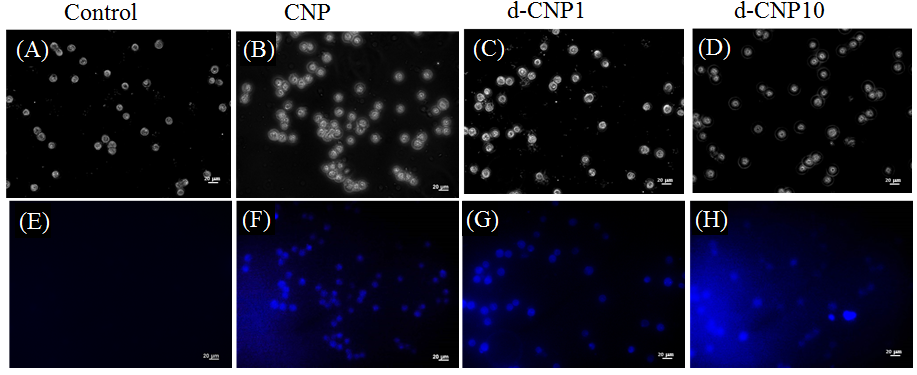


Fig. S9. Confocal fluorescent photos of cells incubation with CNPs under electrical stimulation for 4 hr. Optical photos for (A) control, (B) CNP, (C) d-CNP1 and (D) d-CNP10; and their corresponding fluorescent images for (E) control, (F) CNP, (G) d-CNP1 and (H) d-CNP10. Scale bar: 20 μm.

(3) Detachment and apoptosis of attached cells after endocytosis via a pair of electrified electrodes

The 1 ml L929 fibroblast cells were then seeded onto the 6-well incubation cell-grade dish with 1×10^5^ cell/ml density. Cells without CNPs were used as a blank control. All the cells were incubated at 37±1 ^o^C under 5% CO_2_ blowing for 1 hr for attachment on the dish surfaces. A pair of electrodes connected to a 3 V, 0.7 A power supply was immersed into the cell culture medium (as shown in Fig. S9). The electrical stimulation was performed on pre-attached cells for 1 hr and the situation of cells with different samples was followed via optical microscopy and MTT cell viability tests.

**
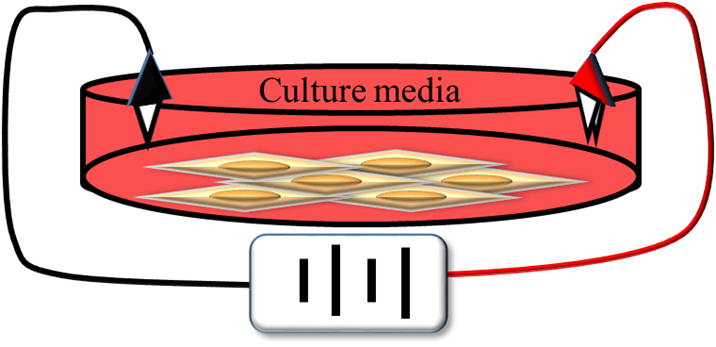
**

Fig. S10. Illustration of electrical stimulation on pre-attached cells.


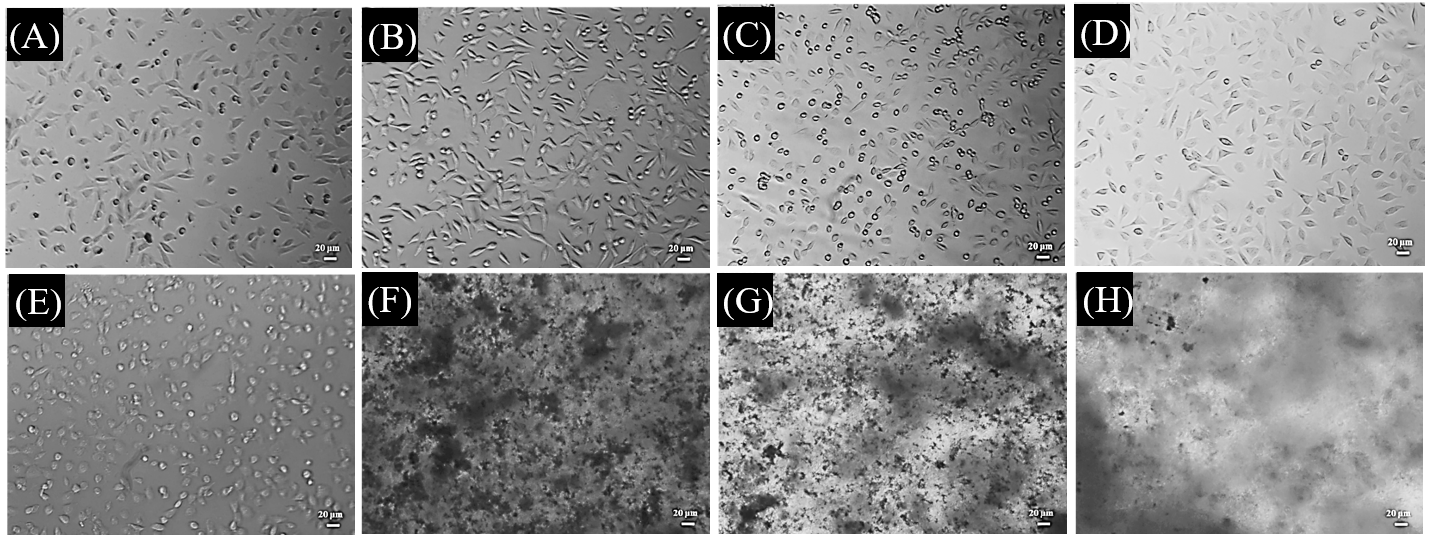


Fig. S11. Optical microphotographs (phase images) for pre-attached electroactive cells before and after 1 hr electrical stimulation with a pair of electrodes with 3 volts and 0.7 amps. (Above) Before electrical stimulation for (A) control, (B) CNP, (C) d-CNP1 and (D) d-CNP10, and (Below) after the stimulation for (E) control, (F) CNP, (G) d-CNP1 and (H) d-CNP10. Scale bar: 20 μm.
